# Therapeutic potential of CD20/CD3 bispecific antibodies in the treatment of autoimmune diseases

**DOI:** 10.1515/rir-2024-0029

**Published:** 2025-01-09

**Authors:** Hongpeng Huang, Xuetao Wei

**Affiliations:** Department of Bioactivity, SinoCellTech Ltd., Beijing 100176, China; Department of Toxicology, School of Public Health, Peking University; Peking China; Beijing Key Laboratory of Toxicological Research and Risk Assessment for Food Safety, Beijing 100191, China

**Keywords:** CD20/CD3 bispecific antibody, autoimmune diseases

## Abstract

Autoimmune diseases arise from immune system dysfunction that immune cells mistakenly attack the body’s own tissues, resulting in systemic disorders or localized lesions such as systemic lupus erythematosus (SLE) and rheumatoid arthritis (RA). Autoreactive B cells play a critical role in the pathogenesis of many autoimmune diseases and B cell depletion using anti-CD20 monoclonal antibody (mAb) has been shown to effectively mitigate disease progression in both preclinical and clinical studies. Recently, bispecific antibody (bsAb) targeting CD20/CD3 have demonstrated substantial clinical benefits in the treatment of various hematologic malignancies. Given their similar B cell cytotoxic mechanism, CD20/CD3 bsAb therapy may offer significant improvements in the management of many autoimmune diseases, providing a novel therapeutic option for patients. This concise review aims to summarize recent findings on CD20/CD3 bsAbs and discuss their potential in treating autoimmune diseases.

## Introduction

Autoimmune diseases are diverse and complex disorders characterized by abnormal immune responses involving both the innate and adaptive immune systems. The intricate pathogenesis and heterogeneity of clinical manifestations complicate their treatment. Despite recent advances in elucidating key pathological processes and understanding crucial molecular functions, significant challenges remain in clinical treatment.^[1]^ Notably, the autoantibodies are thought the most prominent character among the autoimmune diseases. These abnormal antibodies are produced by autoreactive B cells and can attack the body’s own cells and tissues, resulting in organ inflammation and damage. Current primary therapies for autoimmune diseases include immunosuppressive drugs, corticosteroids, and biologics.^[2]^ CD20 is a transmembrane glycoprotein presenting on the surface of B lymphocytes. It is physiologically expressed during the pre-B cell and mature B cell stages, but is absent when B cells differentiate into plasma cells. Pathologically, CD20 is highly expressed in hematologic malignancies such as B-cell-derived lymphomas and leukemias.^[3]^ Numerous studies have demonstrated the involvement of abnormal B cells in the pathogenesis of various autoimmune diseases, highlighting CD20 as a potential target for treating hematologic malignancies and certain autoimmune disorders. Additionally, T cells also play a crucial role in the development of various autoimmune diseases. Preclinical studies have indicated that anti-CD3 therapy can potentially alleviate murine lupus by depleting T cells, inducing T cell anergy, promoting IL-10-secreting regulatory T cells (Treg), and reducing IL-17^+^ follicular helper T cells. These lead to a decrease in autoantibodies and cytokines.^[4,5]^ Unlike the T cell-dependent cytotoxic effects triggered by currently approved CD20/CD3 bsAbs, the therapeutic benefits of anti-CD3 mAbs primarily involve weakening effector T cells and depleting T cells to achieve immune tolerance. While these findings in murine lupus are promising, the clinical efficacy of anti-CD3 mAbs in treating lupus remains uncertain. Notably, Teplizumab, a humanized anti-CD3 mAb, had been approved by the US Food and Drug Administration (FDA) in 2022 to delay the onset of clinical stage 3 type 1 diabetes (T1D) in patients aged 8 years or older with preclinical stage 2 disease.^[6]^ The approval of Teplizumab is largely attributed to lasting immunosuppression and improved β-cell function. Herein, we have outlined the updating data of various CD20/CD3 bsAbs and discussed their therapeutic possibilities in autoimmune diseases.

## CD20-targeted Therapy in Autoimmune Diseases

Rituximab is the first therapeutic anti-CD20 mAb approved for oncology patients in 1997 by FDA of the United States (US). It is recommended to treat nearly all B-cell non-Hodgkin lymphomas (NHLs).^[7]^ Later, as shown in Table 1, Rituximab has been approved for the treatment of many autoimmune diseases, including granulomatosis with polyangiitis, idiopathic thrombocytopenic purpura (ITP), and rheumatoid arthritis (RA).^[8,9]^ Ohter than Rituximab, three anti-CD20 Abs (including Ocrelizumab, Ofatumumab, and Ublituximab) have been approved for the treatment of adult relapsing multiple sclerosis (MS).^[10,11]^ Additionally, Obinutuzumab is undergoing Phase III clinical trials for SLE and lupus nephritis (LN).^[12,13]^ Furthermore, the anti-CD19 antibody Inebilizumab has been approved for the treatment of neuromyelitis optica.^[14]^ Belimumab, as the first approved biologic targeting B-cell activation factor (BAFF) worldwide, is used for the treatment of SLE.^[15]^ Telitacicept, the world’s first recombinant fusion protein targeting both BAFF and a proliferation inducing ligand (APRIL), was approved in China in March 2021 for treating patients with active SLE (Table 1). Additionally, it is under investigation for potential use in treating other autoimmune diseases, including anti-neutrophil cytoplasmic antibody (ANCA)-associated vasculitis, antiphospholipid syndrome, systemic sclerosis (SS), generalized myasthenia gravis, rheumatoid RA, and MS.

**Table 1:** Summary of B cell-targeted therapy in treatment of different autoimmne diseases

Drugs	Companies	Targets	Antibody properties	Approved autoimmune indications
Rituximab	Roche	CD20	Human-mouse Chimeric Antibody	Granulomatosis with polyangiitis, ITP, and RA
Ocrelizumab		CD20	Humanized Antibody	MS
Ofatumumab	Novartis	CD20	Fully Human Antibody	MS
Ublituximab	TG Therapeutics	CD20	Human-mouse Chimeric Antibody	MS
Inebilizumab	Horizon Therapeutics plc	CD19	Humanized Antibody	Neuromyelitis optica
Belimumab	GlaxoSmithKline plc	BAFF	Fully Human Antibody	SLE and LN
Telitacicept	RemeGen	BAFF and APRIL	Recombinant fusion protein	SLE

APRIL, a proliferation-inducing ligand; BAFF, B-cell activating factor; ITP, idiopathic thrombocytopenic purpura; RA, rheumatoid arthritis; MS, multiple sclerosis; SLE, Systemic lupus erythematosus; LN, lupus nephritis.

## The Rationale of B Cell Depletion Therapy in Autoimmune Diseases

The mechanism of action of anti-CD20 mAb therapy in treatment of various autoimmune diseases primarily involves induction of direct apoptosis, antibody-dependent cellular cytotoxicity (ADCC), and complement-dependent cytotoxicity (CDC) to eliminate CD20^+^ cells.^[16]^ This process inhibits their differentiation into plasmablasts and plasma cells, thereby reducing the production of aberrant autoantibodies. In addition, B cells also possess antigen-presenting functions and can activate T cells through specific signaling pathways to regulate inflammatory responses. Hence, treatment with anti-CD20 mAbs interferes with T cell co-stimulatory signals, leading to the inhibition of T cell activation and reduction of inflammatory responses.^[17]^

## Efficacy of anti-CD20 mAb Therapy in Preclinical Studies

As shown in Table 2, numerous preclinical studies have demonstrated the efficacy of targeting CD20 antibodies in treatment of various autoimmune diseases, including LN, autoimmune diabetes, SLE, spontaneous autoimmune thyroiditis (SAT), Graves hyperthyroidism, and RA. Due to species cross-reactivity limitations, the spontaneous diseased mouse models expressing human CD20 proteins are mostly utilized for evaluation of humanized CD20 mAb therapy potency in lupus and autoimmune diabetes. Studies have shown that administration of anti-CD20 mAbs effectively cleared CD20^+^ B cells in peripheral blood, lymph nodes, spleen, bone marrow, and other immune organs. Treatment with anti-CD20 mAbs inhibits T cell activation, reduces levels of various autoantibodies, suppresses inflammation, delays disease progression, and prolongs animal survival. Notably, anti-CD20 mAb therapy combined with oral CD3 treatment displays a significant delay in diabetes development and improves the blood glucose control, which is superior to anti-CD20 mAb monotherapy, suggesting the CD20/CD3 bsAb therapy has great potential in treatment of various autoimmune diseases.

**Table 2:** Summary of in vivo preclinical studies of anti-CD20 therapy or combined therapy in treatment of different autoimmune diseases

Treatment	Mouse strain	Model	Diseases	Results	References
Anti-human CD20 antibody	MRL/MpJ-Fas^lpr^ mice expressing human CD20	Spontaneous	LN	Anti-CD20 antibody treatment was effective to eliminate B cell, improve clinical and pathological disease score, reduce anti nuclear antibody and other autoantibodies	[30]
Anti-human CD20 antibody	NOD mice expressing human CD20	Spontaneous	Autoimmune diabetes	Anti-CD20 antibody treatment depleted B cells in peripheral blood, mesenteric and axillary lymph nodes, and bone marrow. The onset of disease was delayed after anti-CD20 antibody administration.	[31]
Anti-human CD20 antibody combined with anti mouse CD3 antibody	NOD mice expressing human CD20	Spontaneous	Autoimmune diabetes	Anti-CD20 antibody combined with oral anti-CD3 antibody treatment significantly delays diabetes development. The function of regulatory T cells was suppressed after B-cell depletion. Combined therapy was superior to anti-CD20 monotherapy in restoring normoglycemia.	[32]
Anti-mouse CD20 antibody	NZB/NZW F1 mice	Spontaneous	SLE	Anti-CD20 antibody treatment depleted B cells in peripheral blood, spleen, lymph nodes, and bone marrow. When combined with BAFF antibody, further B depletion was observed. The progress of lupus nephritis was notably alleviated after anti-CD20 treatment.	[33]
Anti-mouse CD20 antibody	NZB/NZW F1 mice	Spontaneous	SLE	Anti-CD20 antibody treatment depleted B cells in bone marrow, peripheral blood, spleen, and lymph nodes 7 days after administration, prolonged the survival of disease mice, and mitigated disease progress.	[34]
Obinutuzumab/ Rituximab	MRL/Lpr mice expressing human CD20	Spontaneous	SLE	Obinutuzumab outweighed rituximab in depleting B cells. Obinutuzumab treatment alleviated glomerulus nephritis, decreases anti-RNA antibody levels, reduces the activation of CD4^+^ T cells, and prolong the survival of model mice.	[35]
Anti-mouse CD20 antibody	NOD. H-2 h4 mice	Spontaneous	SAT	Anti-CD20 antibody treatment reduced most B cell in peripheral blood and cervical lymph nodes and nearly 50%–80% of B cells in spleen and reduced thyroglobulin autoantibodies, severity, inflammation, and disease progress.	[36]
Anti-mouse CD20 antibody	BALB/c mice	TSHR A subunit induced	Graves hyperthyroidism	Anti-CD20 antibody treatment eliminated B cells in peripheral blood, spleen and peritoneum, inhibited the increase in IgG levels, IFN-γ, and anti-TSHR antibody, and reduced the development of hyperthyroidism.	[37]
Anti-mouse CD20 antibody	DBA mice	Collagen induced	RA	Anti-CD20 antibody treatment effectively depleted mature B cells in adult DBA-1 mice and delayed in disease onset and autoantibody production, with significantly diminished severity of arthritis both clinically and histologically.	[38]

LN, lupus nephritis; SAT, spontaneous autoimmune thyroiditis; SLE, systemic lupus erythematosus; RA, rheumatoid arthritis; NOD, non-obese diabetic; TSHR, thyroid stimulating hormone receptor; IFN-γ, interferon gamma.

## Limitations of anti-CD20 mAb Therapy in Autoimmune Diseases

In clinical practice, it has been widely acknowledged that certain limitations are existed using anti-CD20 mAb therapies for various autoimmune diseases. Like Rituximab, the chimeric human-mouse antibody-induced immunogenicity causes production of anti-drug antibody (ADA), thereby to some extent reducing its efficacy.^[18]^ Although Rituximab can rapidly deplete peripheral blood B cells, the incapacity to eliminate the long-lived plasma cells in lymph nodes, spleen, and bone marrow has been reported. These plasma cells often lack CD20 expression and highly express CD138 and CD38 instead.^[19]^ Studies have demonstrated that Rituximab can be internalized in the normal B cells and malignant B lymphomas through the FcgRIIb receptor, thereby weakening the FcgR-regulated ADCC effect. Furthermore, elevated levels of BAFF can be observed during repeated Rituximab treatment. As a result, the increased BAFF is capable of restoring B cell function and promoting B cell survival and proliferation.^[20]^ Thus, there is an urgent and unmet need to optimize and develop safer and more effective antibody therapy targeting CD20 for the treatment of various autoimmune diseases.

## Approved CD20/CD3-targeted Therapy

**Figure 1:**
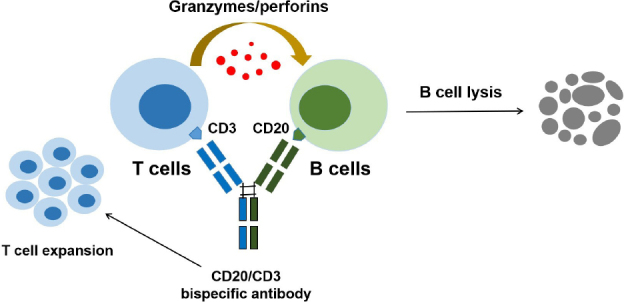
Mechanism of action of CD20/CD3 bsAb-induced B cell lysis. The schematic illustrates CD20/CD3 bsAb simultaneously binding CD20 on the B cells and CD3 on the T cells to induce the cytotoxic effect via effector T cell activation, expansion, and release of multiple cytotoxic factors.

Given the breakthrough progress and excellent clinical trial results in treatment of NHLs, CD20/CD3 bsAbs hold great potential in treating various autoimmune diseases. Currently, three CD20/CD3 bsAbs have been conditionally approved in some countries, including Mosunetuzumab and Glofitamab (Roche), as well as Epcoritamab (AbbVie). As shown in the Table 3. all three bsAbs have been approved in the European (EU) and the US, with Glofitamab being approved not only in the EU and the US but also in China. Currently, Mosunetuzumab is granted priority review, while Epcoritamab is in phase III clinical trials in China. Besides, Odronextamab, GB261, and Plamotamab are also underwent in the clinical trial stage. Unlike the CD20 mAb-induced cytotoxic effects, the two arms of CD20/CD3 bsAbs can simultaneously bind to B cells and T cells, triggering T cell activation and leading to T cell-dependent cellular cytotoxicity (TDCC) against B cells (Figure 1). Based on TDCC, CD20/CD3 bsAbs can effectively eliminate B cells with low CD20 expression levels and induce the B cell death in various immune organs.

**Table 3:** Summary of three approved CD20/CD3 bispecific antibodies

Drugs	Companies	Structure	Indications	Approved location	Approved date	Schedule in China
Mosunetuzumab	Roche	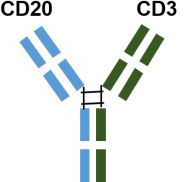	Adult patients with relapsed or refractory follicular lymphoma	EU	2022.06	In November 2023, it was granted priority review for the treatment of adult patients with relapsed or refractory follicular lymphoma who have previously received at least two systemic therapies
		a humanized IgG1 bsAb with 1: 1 CD20: CD3 ratio of Fab arms		US	2022.12	
Glofitamab		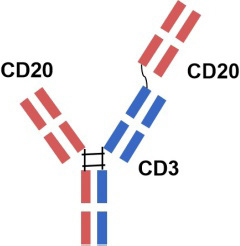	Adult patients with relapsed or refractory diffuse large B-cell lymphoma	Canada	2023.03	/
		a humanized mouse-derived IgG1 bsAb with 2: 1 CD20: CD3 ratio of Fab arms				
				US	2023.06	
				EU	2023.07	
				China	2023.11	
		a humanized IgG1 bsAb against CD20 and CD3				
Epcoritamab	AbbVie	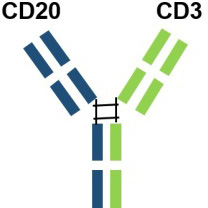	Adult patients with relapsed or refractory diffuse large B-cell lymphoma	US	2023.05	Under Phase III clinical trial for the treatment of patients with relapsed/refractory diffuse large B-cell lymphoma
		with point mutations in the Fab portion of the Fc of the antibody and heterodimerization.		EU	2023.09	

US, the United States; EU, Europe; IgG, Immunoglobin G.

## Efficacy of CD20/CD3 bsAb Therapy in Preclinical Studies

The results of *in vivo* preclinical pharmacodynamic studies of different CD20/CD3 bsAbs are summarized in the Table 4. Mosunetuzumab efficacy was determined using humanized CD20/CD3 mice and human CD34^+^ cells-implanted immune reconstitution model in NSG mice. The results showed that B cells in the periperal blood significantly decreased on days 1 and 7 after intravenous injection of Mosunetuzumab, and the spleen B cells were significantly reduced on 7 days post Mosunetuzumab administration, leading to CD8^+^ T cell activation and expansion in humanized CD20/CD3 mice. In human CD34^+^ cell immune-reconstituted NOD scid gamma mice (NSG) mice, Mosunetuzumab significantly reduced B cells and increased CD8^+^ T cells on days 7, 14, and 21 post-treatment.^[21]^ In a human CD34^+^ cell immune-reconstituted NSG mice subcutaneously implanted with WSU diffuse large B cell lymphoma (DLCL2) tumor cells, Glofitamab induced rapid and complete clearance of B cells in peripheral blood within 24 h after the first administration. After two administrations, complete clearance of B cells in the spleen, bone marrow, and lymph nodes was achieved. This elimination of B cells was accompanied by a transient decrease in T cells. Tumor growth was inhibited post-treatment, with increased intratumoral T cell infiltration.^[22]^ In the humanized NSG mice subcutaneously implanted with Raji cells and human peripheral blood mononuclear cells (PBMCs), a single administration of Epcoritamab significantly inhibited tumor growth, promoted T cell activation, and effectively reduced the number of B cells in peripheral blood.^[23]^ The above-mentioned results have demonstrated that the three marketed CD20/CD3 bsAbs are effective to induce CD20^+^ cell death and inhibit the *in vivo* growth of CD20^+^ tumor cells through T cell activation. Additionally, a single intraperitoneal injection of different doses of Odronextamab showed significant tumor inhibition, demonstrating a dose-response relationship in the NSG mouse subcutaneously implanted with Raji cells and human PBMCs.^[24]^ Likewise, intravenous injection of GB261 rapidly depleted tumor cells through detection of tumor mean fluorescence intensity in the B-NDG mice intravenously injected with rituximab-resistant cell lines (RRCL)-green fluorescent protein (GFP)-firefly luciferase (Luc) cells and human PBMCs.^[25]^ In the humanized CD20/CD3 mice intravenously injected with murine E2A leukemia cells expressing human CD20 protein, a single intravenous injection of Plamotamab significantly reduced the proportion of E2A tumor cells implanted in the bone marrow.^[26]^

**Table 4:** Summary of in vivo preclinical studies of different CD20/CD3 bispecific antibodies

Drugs	Companies	Format	Model	Results	References
Mosunetuzumab	Roche	IgG1	Humanized CD20/CD3 mice	Mosunetuzumab treatment depleted B cells in peripheral blood on on 1 day and 7 days, eliminated B cells in spleen on 7 days, and increased CD8^+^ T cell expansion and activation.	[21]
			NSG mice injected with human CD34^+^ cells	B cell depletion and CD8^+^ T cell expansion were remarkably observed on 7 days, 14 days, and 21 days post Mosunetuzumab administration.	
Glofitamab	Roche	IgG1	NSG mice injected with human CD34^+^ cells subcutaneously implanted with WSU DLCL2 cells	Rapid and complete B cell depletion in peripheral blood was achieved within 24 h after the first dose of Glofitamab. Tthe complete B cell depletion in spleen, bone marrow, and lymph nodes was observed after two doses of Glofitamab, with a transient decline in T cells. Glofitamab significantly induced tumor regression and increased the infiltration of T cells into tumors.	[22]
Epcoritamab	AbbVie	IgG1	Humanized NSG mice subcutaneously implanted Raji cells mixed with human PBMCs at a ratio of 1:1	A single injection of Epcoritamab significantly inhibited tumor growth, promoted T cell activation, and effectively reduced the number of peripheral B cells.	[23]
Odronextamab	Regeneron	IgG4	NSG mice subcutaneously implanted Raji cells mixed with human PBMCs at a ratio of 4:1	A single intraperitoneal injection of Odronextamab at different doses notably inhibited tumor growth in a dose-dependent manner.	[24]
GB 261	Genor Biopharma	IgG1	B-NDG mice intravenously implanted RRCL-GFP-Luc cells with human PBMCs at a ratio of 1:1	Intravenous injection of GB 261 rapidly eliminated tumor cells through the detection of tumor mean fluorescence intensity.	[25]
Plamotamab	Xencor	IgG1	Humanized CD20/CD3 mice intravenously implanted E2A leukemia cells expressing human CD20	A single intravenous injection of Plamotamab significantly reduced E2A leukemia cells implanted in bone marrow.	[26]

DLCL2, diffuse large B cell lymphoma; NSG, NOD scid gamma; PBMCs, peripheral blood mononuclear cells; RRCL, rituximab-resistant cell lines.

## The Therapeutic Potential of CD20/CD3 bsAb Therapy in Autoimmune Diseases

The above-mentioned data have provided the proof-of-concept for the use of CD20/CD3 bsAb therapy in treatment of autoimmune diseases. To date, only two bsAbs have been approved for clinical trials. As shown in Table 5, Mosunetuzumab is currently undergoing a Phase I clinical trial for the treatment of SLE. According to partially disclosed data, several patients treated with Mosunetuzumab experienced sustained peripheral blood B cell depletion, lasting for up to 90 days.

**Table 5:** Summary of CD20/CD3 bispecific antibodies in treatment of different autoimmune diseases in clinical stage

Drugs	Companies	Targets	Format	Indications	Clinical phase	Clinical trial ID
Mosunetuzumab	Roche	CD20/CD3	IgG1	SLE	Phase I	NCT05155345
Imvotamab	IGM Biosciences	CD20/CD3	IgM	SLE	Phase I	NCT06041568
				RA	Phase I	NCT06087406

IgG1, Immunoglobin G1; IgM, Immunoglobin M; SLE, systemic lupus erythematosus; RA, rheumatoid arthritis.

There was also a transient decrease in lymphocytes, which returned to baseline levels within approximately two weeks. Separate patients showed reduced the anti-double-stranded DNA (anti-dsDNA) antibody levels in serum. Overall, no severe treatment-related adverse reactions were observed in patients receiving Mosunetuzumab.

Imvotamab, IgM-based CD20/CD3 bsAbs, is designed to bind more CD20 and CD3 molecules in contrast with the traditional IgG-based bsAbs. It is currently being evaluated in Phase I clinical trials for the treatment of SLE and RA. Preclinical studies indicated that Imvotamab had a strong affinity for human CD20 and induced TDCC more effectively than CDC. It also exhibited strong cytotoxicity against Rituximab-resistant Ramos cells. *In vivo* efficacy studies indicated that Imvotamab significantly inhibited the subcutaneous growth of Raji cells in the tumor-bearing mice, achieving a tumor inhibition rate of 83% and significantly extending the survival of experimental animals. Moreover, preclinical studies in cynomolgus monkeys revealed that a single injection of Imvotamab effectively depleted peripheral blood B cells through both TDCC and CDC.

## Safety Concerns of CD20/CD3 bsAb Therapy in Autoimmune Diseases

In clinical trials, the most common adverse events associated with CD20/CD3 bsAb therapy are related to cytokine release syndrome (CRS). CRS is characterized by symptoms such as chills, fever, nausea, fatigue, hypotension, hypoxia, confusion, and body aches, some of which result from the over-activation of T cells. According to the recent reports, the incidence of CRS varies among the three CD20/CD3 bsAbs, with a high occurrence of Grade 1 CRS (26% to 47%). No cases of Grade 5 CRS were reported during CD20/CD3 bsAb therapy. The median duration of CRS was 3 days for Mosunetuzumab, 2 days for Epcoritamab, and 30.5 h for Glofitamab. Neurological toxicity was reported less frequently and typically at lower grades.^[27]^ Other adverse events, such as neutropenia, hypophosphatemia, anemia, and diarrhea, were also observed across different clinical studies, but these were mostly Grade 1 or 2 and reversible.^[28]^ Overall, the safety profile of CD20/CD3 bsAbs in treating hematologic malignancies has been generally manageable, and therapy discontinuation due to adverse effects was rare.^[29]^ Currently, data on the use of CD20/CD3 bsAbs for treating autoimmune diseases is limited. In patients with SLE, CD20/CD3 bsAbs caused a temporary reduction in lymphocyte levels, which rapidly returned to baseline. Preliminary clinical data indicate a favorable safety profile for SLE patients following CD20/ CD3 bsAb administration. However, it is important to note that the safety data after long-term B cell depletion are still lacking, and there is a need to monitor the potential adverse events related to delayed B-cell recovery.

## Conclusions and Perspectives

Based on the outcomes of preclinical and clinical trials CD20/ CD3 bsAbs exhibit a potent T cell-mediated cytotoxic mechanism, leading to extensive and effective B cell depletion. This mechanism inhibits B cell differentiation into plasma cells, reduces autoantibody production, decreases immune complex formation, and suppresses inflammatory responses, thereby ameliorating clinical manifestations and delaying disease progression and relapse. Notably, most CD20/CD3 bsAbs, with the exception of Odronextamab (IgG4-based format), are designed using a mutated IgG1 format. It can circumvent antibody-dependent FcYR-mediated cross-binding of CD3 and T cells, preventing antigen-independent T-cell activation and cytotoxicity, as well as fratricidal ADCC and CDC. Interestingly, GB261 is an exception and still retains ADCC and CDC functions specific to CD20 cells. It is engineered with low CD3 binding affinity to mitigate the risk of CRS, a significant safety concern in CD3-based bsAb treatments. Additionally, most IgG-based CD20/CD3 bsAbs are formatted with a 1: 1 ratio of CD20 to CD3. However, Glofitamab, with a 2: 1 ratio of CD20 and CD3 ratio, is thought to possess a stronger CD20-binding capacity. The structural design and binding affinity variations among bsAbs may influence their efficacy in treating autoimmune diseases. In addition to CRS, another concern impacting CD20/CD3 bsAb efficacy is T cell function. Immunosuppressant-treated patients typically have compromised T cell function, potentially affecting the TDCC effect. Whether CD20/CD3 bsAb therapy is effective to those patients who have a compromised T cell function after immunossuppressant treatment is still required investigation. Despite little understanding with regards to CD20/ CD3 bsAbs in treating autoimmune diseases, these bsAbs hold promise as monotherapy, possibly combined with anti-CD20 therapy, or for anti-CD20 resistant conditions. Further investigation would also focus on combining CD20/CD3 bsAb therapy with standard therapy for specific autoimmune diseases. In conclusion, novel CD20/CD3 bsAbs represent a promising therapeutic option for patients with autoimmune diseases.
